# 6,8-Dibromo­quinoline

**DOI:** 10.1107/S1600536810043242

**Published:** 2010-10-31

**Authors:** Ísmail Çelik, Mehmet Akkurt, Osman Çakmak, Salih Ökten, Santiago García-Granda

**Affiliations:** a Department of Physics, Faculty of Arts and Sciences, Cumhuriyet University, 58140 Sivas, Turkey; b Department of Physics, Faculty of Sciences, Erciyes University, 38039 Kayseri, Turkey; c Department of Chemistry, Faculty of Arts and Sciences, Gaziosmanpaşa University, 60240 Tokat, Turkey; d Departamento Química Física y Analítica, Facultad de Química, Universidad Oviedo, C/ Julián Clavería, 8, 33006 Oviedo (Asturias), Spain

## Abstract

The title mol­ecule, C_9_H_5_Br_2_N, is almost planar, with an r.m.s. deviation of 0.027 Å. The dihedral angle between the aromatic rings is 1.5 (3)°. In the crystal, π–π stacking inter­actions are present between the pyridine and benzene rings of adjacent mol­ecules [centroid–centroid distances = 3.634 (4) Å], and short Br⋯Br contacts [3.4443 (13) Å] occur.

## Related literature

For the biological and pharmacological activities of quinolines and their derivatives, see: Abadi *et al.* (2005 ▶); Blackie *et al.* (2007 ▶); Chen *et al.* (2006 ▶); Gómez *et al.* (2008 ▶); Gómez-Barrio *et al.* (2006 ▶); Kouznetsov *et al.* (2005 ▶, 2007 ▶); Lindley (1984 ▶); Metwally *et al.* (2006 ▶); Muscia *et al.* (2006 ▶); Musiol *et al.* (2007 ▶); Sissi & Palumbo (2003 ▶); Vangapandu *et al.* (2004 ▶); Vinsova *et al.* (2008 ▶); Vladímir *et al.* (2005 ▶); Zhao *et al.* (2005 ▶); Zhu *et al.* (2007 ▶); Şahin *et al.* (2008 ▶). For the synthesis, see: Ökten *et al.* (2010 ▶).
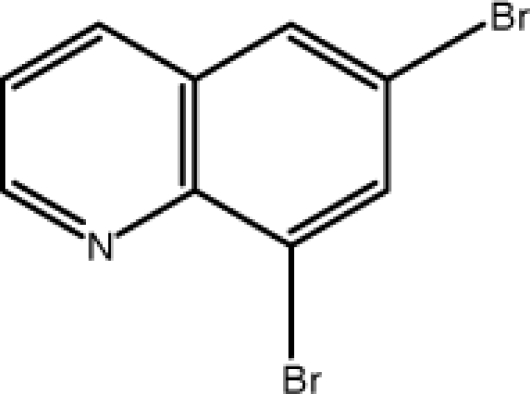


## Experimental

### 

#### Crystal data

C_9_H_5_Br_2_N
*M* *_r_* = 286.94
Monoclinic, 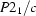

*a* = 7.3436 (12) Å
*b* = 9.8961 (15) Å
*c* = 13.0108 (18) Å
β = 109.589 (17)°
*V* = 890.8 (3) Å^3^
*Z* = 4
Cu *K*α radiation
μ = 11.04 mm^−1^
*T* = 297 K
0.12 × 0.09 × 0.02 mm

#### Data collection

Oxford Diffraction Xcalibur diffractometer with a Ruby Gemini CCD detector
Absorption correction: part of the refinement model (Δ*F*) (*XABS2*; Parkin *et al.*, 1995 ▶)*T*_min_ = 0.052, *T*_max_ = 0.080
1598 measured reflections
1598 independent reflections
1075 reflections with *I* > 2σ(*I*)

#### Refinement

*R*[*F* ^2^ > 2σ(*F* ^2^)] = 0.045
*wR*(*F* ^2^) = 0.141
*S* = 1.02
1598 reflections
109 parameters
H-atom parameters constrained
Δρ_max_ = 0.68 e Å^−3^
Δρ_min_ = −0.56 e Å^−3^

### 

Data collection: *CrysAlis PRO* (Oxford Diffraction, 2009 ▶); cell refinement: *CrysAlis PRO*; data reduction: *CrysAlis PRO*; program(s) used to solve structure: *SIR97* (Altomare *et al.*, 1999 ▶); program(s) used to refine structure: *SHELXL97* (Sheldrick, 2008 ▶); molecular graphics: *ORTEP-3 for Windows* (Farrugia, 1999 ▶); software used to prepare material for publication: *WinGX* (Farrugia, 1997 ▶) and *PLATON* (Spek, 2009 ▶).

## Supplementary Material

Crystal structure: contains datablocks global, I. DOI: 10.1107/S1600536810043242/hb5698sup1.cif

Structure factors: contains datablocks I. DOI: 10.1107/S1600536810043242/hb5698Isup2.hkl

Additional supplementary materials:  crystallographic information; 3D view; checkCIF report

## Figures and Tables

**Fig. 1.**
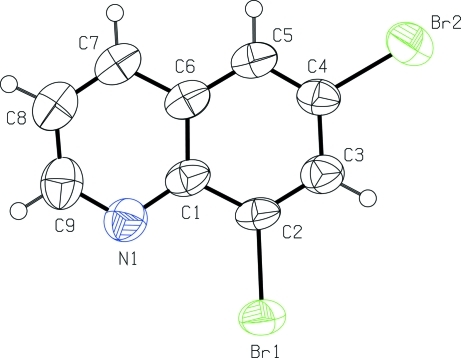
The title molecule with displacement ellipsoids for non-H atoms drawn at the 50% probability level.

**Fig. 2.**
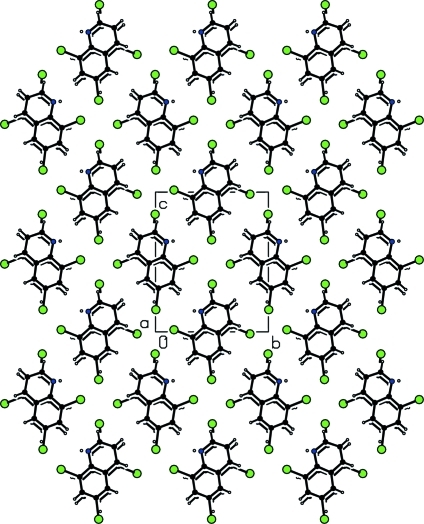
View of the packing of (I) down the a axis.

## supplementary crystallographic information

### Comment

The quinoline skeleton is often used for designing of many synthetic compounds with diverse pharmacological and medicinal properties. Quinolines and their derivatives have shown to display a wide spectrum of biological activities such as antibacterial (Metwally *et al.*, 2006), antimycobacterial (Vinsova *et al.*, 2008; Vangapandu *et al.*, 2004), antineoplastic (Zhao *et al.*, 2005; Sissi & Palumbo, 2003; Musiol *et al.*, 2007; Zhu *et al.*, 2007), antiparasitical (Muscia *et al.*, 2006; Blackie *et al.*, 2007; Gómez *et al.*, 2008; Gómez-Barrio *et al.*, 2006; Kouznetsov *et al.*, 2005, 2007), and anti-inflammatory behavior (Chen *et al.*, 2006; Abadi *et al.*, 2005; Ökten *et al.*, 2010). Quinoline also constitutes a key structural component of numerous compounds with pharmacological activity, dyestuffs, materials with metal-halogen exchange, and agrochemical (Lindley, 1984) and couplings (Vladímir *et al.*, 2005). Bromoquinolines have been of interest for chemists as precursors for heterocyclic compounds due to important scaffolds in medicinal chemistry. It was developed a convenient synthetic methodology for 6,8-disubstituted quinoline derivatives and the values of 6,8-dibromoquinoline as precursors to the corresponding disubstituted quinolines were presented. New disubstituted quinoline derivatives were synthesized *via* substition reaction by using 6,8-DiBrQ, converted to further substituted quinoline. That may serve for the synthesis of natural bioactive quinolines derivatives because there are many biological active 6 and 8- functionalized quinolines such as quinine, pentaquine, and plasmoquine (Şahin *et al.*, 2008).

The molecular structure of the title compound (I) is shown in Fig. 1 with their respective labels. Bond lengths and angles in (I) are within normal ranges. In this structure, the quinoline motif (N1/C1–C9) is essentially planar with maxium deviations of 0.029 (7) Å for C3 and 0.031 (9) Å for C8. The Br1—C2—C3—C4 and Br2—C4—C5—C6 torsion angles are -179.0 (5) and 178.7 (5)°, respectively.

The crystal structure of (I) is stabilized by π–π stacking interactions, along the *a* axis, between N1/C1/C6–C9 (centroid *Cg*1) and C1–C6 (centroid *Cg*2) rings, with a *Cg*1···*Cg*2 distance of 3.634 (4) Å, (Fig. 2).

### Experimental

6,8-DiBromo-1,2,3,4-tetrahydroquinoline was synthesized in proper literature (Ökten *et al.*, 2010). Then, DDQ (2 g, 6.88 mmol) was dissolved in freshly distilled and dried bezene (10 ml) under an argon atmosphere. To a solution of 6,8-diBrTHQ (1 g, 3.44 mmol) in benzene (30 ml) was added the solution of DDQ. The mixture was refluxed at 353 K for 36 h. Upon cooling, the dark green solidified mixture was filtered and the solvent was removed in vacuo. The residue was filtered from a short silica column (1/9, EtOAc/hexane, *R_f_*= 0.4). Recrystallization of the product from hexane–chloroform gave 6,8-diBrQ in a yield of 88% (868 mg) as colourless plates, m.p. 372–373 K. ^1^H NMR (CDCl_3_, 400 MHz) d 9.04 (dd, *J_23_*= 4.2 Hz, *J_24_*= 1.6 Hz, 1H, H_2_), 8.16 (d, *J_57_*= 2.4 Hz, 1H, H_7_), 8.09 (dd, *J_43_*= 8.3 Hz, *J_24_*= 1.6 Hz, 1H, H_4_), 7.96 (d, *J_57_*= 2.4 Hz, 1H, H_5_), 7.49 (dd, *J_32_*= 4.2 Hz, *J_34_*= 8.3 Hz, 1H, H_3_); ^13^C NMR (100 MHz, CDCl_3_) d 151.5, 144.1135.9, 135.7, 130.1, 129.7, 125.9, 122.7, 119.9; IR (KBr, cm^-1^) *v*max 3026, 1638, 1617, 1587, 1545, 1467, 1443, 1347, 1306, 1183, 1084, 1030, 962, 857, 809, 779, 677, 593, 543, 501. GC–MS *m/z* 289 (5, *M*^+^), 288 (50), 287 (10), 286 (98), 285 (10), 284 (42), 207 (30), 205 (31), 129 (5), 127 (10), 126 (100), 125 (14), 103 (15), 102 (14), 99 (37), 98 (33), 97 (20), 75 (19), 74 (22), 73 (42), 50 (18), 49 (52), 48 (14), 37 (7), 36 (7). Anal. Calcd for C_9_H_5_NBr_2_ (286.95): C 37.67, H 1.76%. Found: C 37.78, H 1.82%.

### Refinement

H atoms were included in geometric positions with C—H = 0.93 Å and refined by using a riding model [*U*_iso_(H) = 1.2*U*_eq_(C)].

### Crystal data

**Table:**

C_9_H_5_Br_2_N	*F*(000) = 544
*M**_r_* = 286.94	*D*_x_ = 2.140 Mg m^−^^3^
Monoclinic, *P*2_1_/*c*	Cu *K*α radiation, λ = 1.54184 Å
Hall symbol: -P 2ybc	Cell parameters from 1247 reflections
*a* = 7.3436 (12) Å	θ = 3.6–70.3°
*b* = 9.8961 (15) Å	µ = 11.04 mm^−^^1^
*c* = 13.0108 (18) Å	*T* = 297 K
β = 109.589 (17)°	Plate, colourless
*V* = 890.8 (3) Å^3^	0.12 × 0.09 × 0.02 mm
*Z* = 4	

### Data collection

**Table:**

Oxford Diffraction Xcalibur diffractometer with a Ruby Gemini CCD detector	1598 independent reflections
Radiation source: Enhance (Cu) X-ray Source	1075 reflections with *I* > 2σ(*I*)
graphite	*R*_int_ = 0.0000
ω scans	θ_max_ = 70.5°, θ_min_ = 5.8°
Absorption correction: part of the refinement model (Δ*F*) [*XABS2* (Parkin *et al.*, 1995); cubic fit to sin(θ)/λ - 24 parameters]	*h* = −8→8
*T*_min_ = 0.052, *T*_max_ = 0.080	*k* = 0→11
1598 measured reflections	*l* = 0→15

### Refinement

**Table:**

Refinement on *F*^2^	Primary atom site location: structure-invariant direct methods
Least-squares matrix: full	Secondary atom site location: difference Fourier map
*R*[*F*^2^ > 2σ(*F*^2^)] = 0.045	Hydrogen site location: inferred from neighbouring sites
*wR*(*F*^2^) = 0.141	H-atom parameters constrained
*S* = 1.02	*w* = 1/[σ^2^(*F*_o_^2^) + (0.0642*P*)^2^] where *P* = (*F*_o_^2^ + 2*F*_c_^2^)/3
1598 reflections	(Δ/σ)_max_ < 0.001
109 parameters	Δρ_max_ = 0.68 e Å^−^^3^
0 restraints	Δρ_min_ = −0.56 e Å^−^^3^

### Special details

**Table:**

Geometry. Bond distances, angles *etc*. have been calculated using the rounded fractional coordinates. All su's are estimated from the variances of the (full) variance-covariance matrix. The cell e.s.d.'s are taken into account in the estimation of distances, angles and torsion angles
Refinement. Refinement on *F*^2^ for ALL reflections except those flagged by the user for potential systematic errors. Weighted *R*-factors *wR* and all goodnesses of fit *S* are based on *F*^2^, conventional *R*-factors *R* are based on *F*, with *F* set to zero for negative *F*^2^. The observed criterion of *F*^2^ > σ(*F*^2^) is used only for calculating -*R*-factor-obs *etc*. and is not relevant to the choice of reflections for refinement. *R*-factors based on *F*^2^ are statistically about twice as large as those based on *F*, and *R*-factors based on ALL data will be even larger.

### Fractional atomic coordinates and isotropic or equivalent isotropic displacement parameters (Å^2^)

**Table:**

	*x*	*y*	*z*	*U*_iso_*/*U*_eq_	
Br1	0.93324 (12)	0.16374 (8)	0.01474 (6)	0.0736 (3)	
Br2	0.54665 (13)	0.49672 (10)	−0.34185 (6)	0.0832 (4)	
N1	0.8781 (8)	0.4050 (6)	0.1454 (4)	0.0641 (19)	
C1	0.8034 (8)	0.4311 (7)	0.0368 (5)	0.055 (2)	
C2	0.8120 (8)	0.3287 (6)	−0.0384 (5)	0.0528 (19)	
C3	0.7418 (9)	0.3499 (7)	−0.1488 (5)	0.0595 (19)	
C4	0.6545 (9)	0.4739 (7)	−0.1875 (5)	0.0550 (19)	
C5	0.6420 (9)	0.5744 (7)	−0.1208 (5)	0.059 (2)	
C6	0.7184 (9)	0.5558 (7)	−0.0071 (5)	0.058 (2)	
C7	0.7125 (10)	0.6584 (8)	0.0673 (6)	0.067 (3)	
C8	0.7919 (11)	0.6338 (9)	0.1768 (6)	0.075 (3)	
C9	0.8687 (11)	0.5055 (9)	0.2115 (6)	0.075 (3)	
H3	0.75210	0.28330	−0.19700	0.0710*	
H5	0.58330	0.65570	−0.14950	0.0710*	
H7	0.65560	0.74140	0.04220	0.0800*	
H8	0.79470	0.70100	0.22730	0.0900*	
H9	0.91680	0.48940	0.28620	0.0900*	

### Atomic displacement parameters (Å^2^)

**Table:**

	*U*^11^	*U*^22^	*U*^33^	*U*^12^	*U*^13^	*U*^23^
Br1	0.0902 (6)	0.0440 (5)	0.0739 (5)	0.0092 (4)	0.0106 (4)	0.0044 (3)
Br2	0.1024 (7)	0.0722 (7)	0.0649 (5)	0.0056 (5)	0.0146 (4)	0.0115 (4)
N1	0.065 (3)	0.056 (4)	0.068 (3)	−0.007 (3)	0.018 (2)	−0.005 (3)
C1	0.047 (3)	0.040 (4)	0.074 (4)	−0.003 (3)	0.014 (3)	−0.004 (3)
C2	0.052 (3)	0.033 (4)	0.070 (3)	0.001 (3)	0.016 (3)	0.005 (3)
C3	0.057 (3)	0.049 (4)	0.066 (3)	−0.001 (3)	0.012 (3)	−0.001 (3)
C4	0.056 (3)	0.046 (4)	0.061 (3)	−0.001 (3)	0.017 (3)	0.009 (3)
C5	0.055 (3)	0.043 (4)	0.076 (4)	0.004 (3)	0.019 (3)	0.002 (3)
C6	0.050 (3)	0.047 (4)	0.078 (4)	0.001 (3)	0.024 (3)	−0.004 (3)
C7	0.067 (4)	0.052 (5)	0.084 (4)	0.005 (3)	0.030 (3)	−0.007 (4)
C8	0.079 (5)	0.068 (6)	0.084 (5)	−0.004 (4)	0.036 (4)	−0.013 (4)
C9	0.077 (5)	0.078 (6)	0.070 (4)	−0.016 (4)	0.025 (3)	−0.015 (4)

### Geometric parameters (Å, °)

**Table:**

Br1—C2	1.877 (6)	C5—C6	1.407 (9)
Br2—C4	1.909 (6)	C6—C7	1.414 (10)
N1—C1	1.358 (8)	C7—C8	1.368 (10)
N1—C9	1.332 (10)	C8—C9	1.401 (12)
C1—C2	1.425 (9)	C3—H3	0.9300
C1—C6	1.414 (10)	C5—H5	0.9300
C2—C3	1.370 (9)	C7—H7	0.9300
C3—C4	1.398 (10)	C8—H8	0.9300
C4—C5	1.343 (9)	C9—H9	0.9300
			
Br1···Br1^i^	3.4443 (13)		
			
C1—N1—C9	116.1 (6)	C5—C6—C7	122.2 (6)
N1—C1—C2	118.9 (6)	C6—C7—C8	119.0 (7)
N1—C1—C6	123.8 (6)	C7—C8—C9	118.8 (7)
C2—C1—C6	117.3 (6)	N1—C9—C8	124.8 (7)
Br1—C2—C1	119.4 (5)	C2—C3—H3	121.00
Br1—C2—C3	119.0 (5)	C4—C3—H3	121.00
C1—C2—C3	121.6 (6)	C4—C5—H5	120.00
C2—C3—C4	118.6 (6)	C6—C5—H5	120.00
Br2—C4—C3	117.5 (5)	C6—C7—H7	120.00
Br2—C4—C5	119.9 (5)	C8—C7—H7	120.00
C3—C4—C5	122.7 (6)	C7—C8—H8	121.00
C4—C5—C6	119.5 (6)	C9—C8—H8	121.00
C1—C6—C5	120.3 (6)	N1—C9—H9	118.00
C1—C6—C7	117.5 (6)	C8—C9—H9	118.00
			
C9—N1—C1—C2	178.9 (6)	C1—C2—C3—C4	−2.1 (10)
C9—N1—C1—C6	−0.3 (10)	C2—C3—C4—C5	2.1 (11)
C1—N1—C9—C8	−1.3 (12)	C2—C3—C4—Br2	−176.9 (5)
N1—C1—C2—Br1	−2.1 (8)	Br2—C4—C5—C6	178.7 (5)
N1—C1—C2—C3	−179.0 (6)	C3—C4—C5—C6	−0.3 (11)
C6—C1—C2—C3	0.3 (9)	C4—C5—C6—C7	179.0 (7)
N1—C1—C6—C5	−179.2 (6)	C4—C5—C6—C1	−1.6 (10)
C6—C1—C2—Br1	177.2 (5)	C1—C6—C7—C8	1.4 (11)
C2—C1—C6—C5	1.6 (10)	C5—C6—C7—C8	−179.2 (7)
C2—C1—C6—C7	−179.0 (6)	C6—C7—C8—C9	−2.8 (12)
N1—C1—C6—C7	0.2 (10)	C7—C8—C9—N1	2.9 (13)
Br1—C2—C3—C4	−179.0 (5)		

Symmetry codes: (i) −*x*+2, −*y*, −*z*.
